# Observation of a Metastable Honeycomb Arrangement of C_60_ on Ni(111) with (7 × 7) Periodicity: Tailoring an Interface for Organic Spintronics

**DOI:** 10.1021/acsanm.1c02060

**Published:** 2021-12-07

**Authors:** Andrea Picone, Marco Finazzi, Lamberto Duò, Dario Giannotti, Franco Ciccacci, Alberto Brambilla

**Affiliations:** Dipartimento di Fisica, Politecnico di Milano, Piazza Leonardo da Vinci, 32, Milano 20133, Italy

**Keywords:** fullerenes, nickel, spinterfaces, surface structure, scanning tunneling microscopy

## Abstract

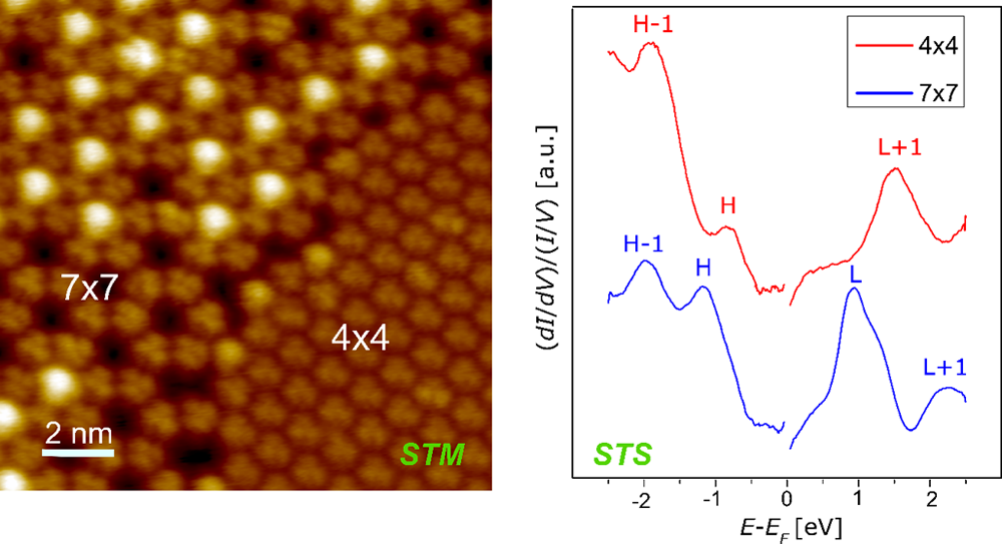

Hybrid nanostructures in which organic molecules are interfaced with metal surfaces hold promise for the discovery of intriguing physical and chemical phenomena, as well as for the development of innovative devices. In this frame, it is crucial to understand the interplay between the structural details of the interface and the electronic properties of the system. Here, an experimental investigation of the C_60_/Ni(111) interface is performed by means of scanning tunneling microscopy/spectroscopy (STM/STS) and low-energy electron diffraction (LEED). The deposition of C_60_ at room temperature, followed by high-temperature annealing, promotes the stabilization of two different phases. A hitherto unreported phase forming a (7 × 7) honeycomb overlayer coexists with the well-known (4 × 4) reconstruction. Highly resolved STM images disclose the adsorption geometry of the molecules for both phases. STS reveals that the electronic properties of C_60_/Ni(111) are strongly influenced by the morphology of the interface, suggesting the possibility of tuning the electronic properties of the organic/inorganic heterostructures by adjusting the structural coupling with the substrate. This achievement can be important for hybrid magnetic interfaces, where the harmonization between the molecular and the magnetic orders can enhance the development of hybrid magnetic states.

## 1 Introduction

During the past decades, ordered overlayers of C_60_ fullerene molecules self-assembled on metallic substrates have been the subject of many theoretical and experimental studies.^1−4^ Such investigations have been motivated by the need to understand the fundamental physical and chemical properties of such systems as well as by the increasing interest in several possible applications that could be derived from them.^5−9^ Fullerenes are indeed currently employed in electronics, photovoltaics, and medical applications, also because of the opportunities offered by either doping or functionalizing the molecules.^10−12^

Furthermore, interfaces between organic semiconductors and ferromagnetic metals have also been investigated in connection with organic spintronics, which have, in more recent years, produced several intriguing results of new magnetic effects at those so-called spinterfaces.^13,14^ In particular, several studies have demonstrated that C_60_ is well-suited both for the fabrication of benchmark spintronics devices, such as spin valves,^15,16^ and for the realization of novel spin-polarized hybrid interface states, which can be induced either in the molecular layer^17^ or even in a nonmagnetic substrate, like Cu.^18^

Among the intriguing aspects related to C_60_/metal interfaces, it is worth mentioning that the molecular adsorption on low-index metallic surfaces generally induces a noticeable restructuring of the substrate.^19−21^ This, in turn, suggests that fullerene molecules can be used to modify and possibly tailor interfacial properties such as, in particular, spin-polarized states and magnetic moments.^22^

Atomic and electronic structures have been studied and determined for C_60_ films grown on various substrates, such as Fe, Co, Pt, and Au, just to name a few.^23−26^ In particular, (111) metallic surfaces are well suited for the stabilization of ordered C_60_ overlayers. Several molecular superstructures have indeed been observed, such as  on Au(111),^27−29^ (4 × 4) on Cu(111),^30,31^ on Pt(111).^20,32^ On the other hand, only few studies have been devoted to the investigation of C_60_ interfaced with Ni(111).^33−35^ They show that C_60_ generally forms an epitaxial layer, with a (4 × 4) superstructure. Recent theoretical calculations, by Pang and co-workers, suggest that this phase involves the reconstruction of the Ni(111) surface layer, with the formation of holes in the substrate in which the molecules are accommodated.^22^ The electronic and magnetic properties of the reconstructed phase are predicted to be different with respect to the case in which C_60_ is simply adsorbed on the Ni(111) topmost layer.^22^

In this paper, we report on a new phase that characterizes the arrangement of C_60_ in self-assembled monolayers on Ni(111), and that has not been observed and discussed so far. In particular, deposition of C_60_ on the Ni(111) surface kept at room temperature (RT) followed by annealing at temperatures of about 400 °C leads to the stabilization of a honeycomb molecular reconstruction, in which the hollow sites are found to be either empty or filled by single C_60_ molecules, resulting in a (7 × 7) superstructure. The (7 × 7) phase coexists with the well-known (4 × 4) reconstruction. Annealing at temperatures higher than 400 °C eventually leads to the stabilization of the (4 × 4) phase all over the surface, suggesting that the (7 × 7) phase is metastable. Finally, we also observe that the electronic structure of the two phases differs significantly close to the Fermi level, likely on account of the different hybridization with the substrate.

## 2 Experimental Methods

Ni(111) single crystal substrates were prepared in ultrahigh vacuum (UHV) conditions (base pressure in the 10^–8^ Pa range) via repeated cycles of Ar^+^ ion sputtering (*I* = 1.5 μA, *V* = 1.5 kV) and thermal annealing at *T* = 500 °C, as in previous experiments.^36^ The sample temperature was measured by a thermocouple mounted in close proximity to the sample position.

Fullerene films were grown *in situ* from an outgassed Ta crucible, under the same UHV conditions, with a typical growth rate of about 0.03 equivalent monolayers (ML) per minute, where 1 ML equals the amount of C_60_ molecules needed to form a single layer of hexagonal close-packed fullerene, i.e., about 1.2 molecules/nm^2^. Notice that the mentioned packing corresponds to a (4 × 4) superstructure, as discussed below. The growth rate was calibrated by a quartz microbalance. The substrates were kept at RT during fullerene deposition.

Scanning tunneling microscopy (STM) and spectroscopy (STS) were performed by using an Omicron variable temperature STM in a UHV chamber connected with the preparation system. Images were always acquired at RT in constant-current mode with homemade electrochemically etched W tips. The bias voltage reported for each measurement is referred to the sample.

## 3 Results and Discussion

**Figure 1:**
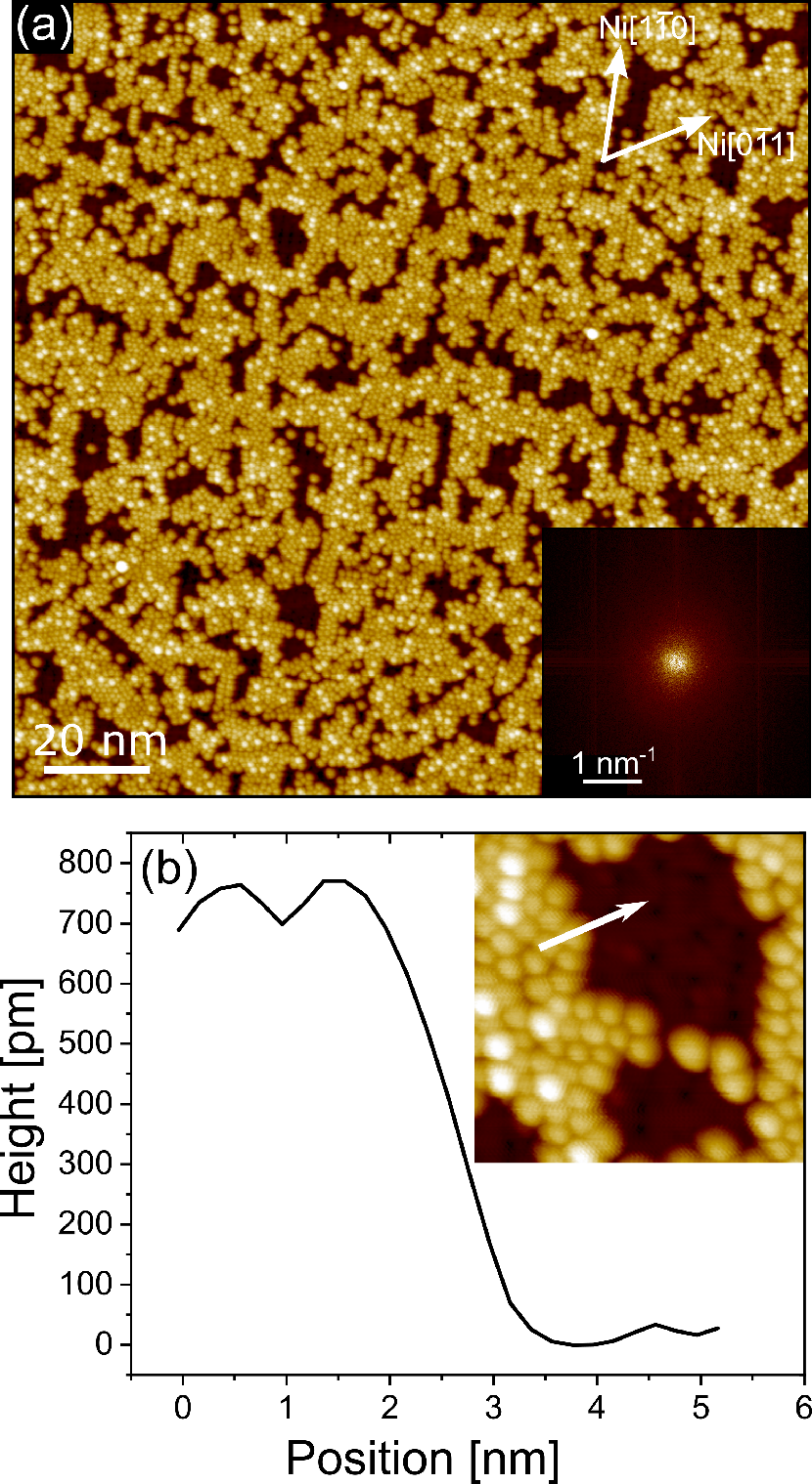
(a) STM topography image of 0.8 ML C_60_ as grown on the Ni(111) surface at RT (tunneling parameters Δ*V* = 1.5 V, *I* = 400 pA). Inset: Fast Fourier transform (absolute value) of the STM image. (b) Topographic profile along the white arrow drawn in the zoomed image reported in the same panel.

The early stages of growth of C_60_ on the Ni(111) surface are illustrated in Figure 1. The substrate surface (image not shown) is characterized by atomically flat terraces separated by monatomic steps. Once fullerene is evaporated with the sample kept at RT, the growing layer consists of a disordered film in the submonolayer regime, as shown in Figure 1a. The lack of long-range order in the molecular layer is evidenced by the absence of spots in the fast Fourier transform (FFT) of the STM image, whose absolute value is shown in the inset of Figure 1a. The stabilization of a disordered overlayer suggests that, on the Ni(111) surface, C_60_ experiences a high diffusion barrier, which reduces the fullerene RT mobility, as already observed on other transition metal surfaces.^8^ The line profile reported in Figure 1b, crossing a border between a C_60_ island and the Ni(111) substrate, is characterized by a step height of about 700 pm, well consistent with the molecular diameter, as reported in other cases.^8,17^

**Figure 2:**
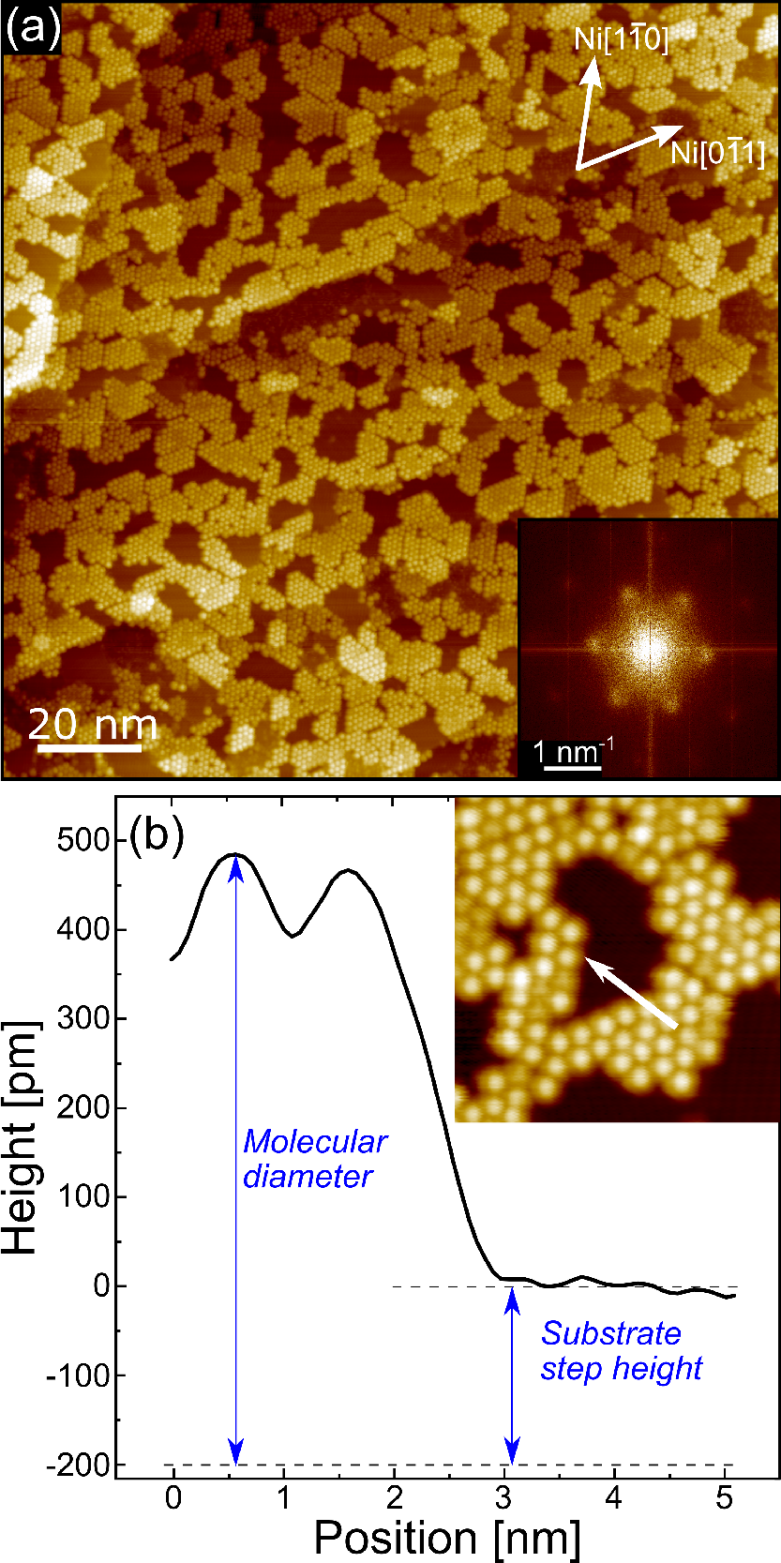
(a) STM topography image of 0.8 ML C_60_ grown on the Ni(111) surface at RT and then annealed at *T* = 400 °C (tunneling parameters Δ*V* = 1.7 V, *I* = 400 pA). Inset: Fast Fourier transform (absolute value) of the STM image. (b) Topographic profile along the white arrow drawn in the zoomed image reported in the same panel. The double arrow blue lines drawn under the profile can be used for comparing the molecular diameter to the Ni(111) step height, as discussed in the text.

When the 0.8 ML C_60_ layer is annealed at *T* = 400 °C for 5 min, it eventually reaches an ordered arrangement, characterized by the formation of small islands, as seen in the STM image reported in Figure 2a and from the corresponding FFT shown in the inset. The hexagonal pattern visible in the FFT image corresponds to a (4 × 4) superstructure. According to recent theoretical investigations, the stabilization of the C_60_ (4 × 4) superstructure involves, underneath each fullerene molecule, the displacement of seven Ni atoms from the topmost Ni layer.^22^ The molecules are accommodated inside the resulting cavity; therefore the topographic height expected for C_60_ molecules onto the Ni(111) surface would be, in that case, lower than the molecular diameter of 700 pm. Such a reduction of the C_60_ height is indeed observed in the annealed sample, in agreement also with the observations of ref (33). The line profile reported in Figure 2b reveals, in fact, a C_60_ height slightly larger than 400 pm. Considering that the interlayer spacing in Ni(111) is about 200 pm, the topography is consistent with a molecule embedded between the first and second layers of the substrate. Such a scheme is exemplified by reporting the molecular diameter and the Ni(111) step height under the line profile of Figure 2b.

**Figure 3:**
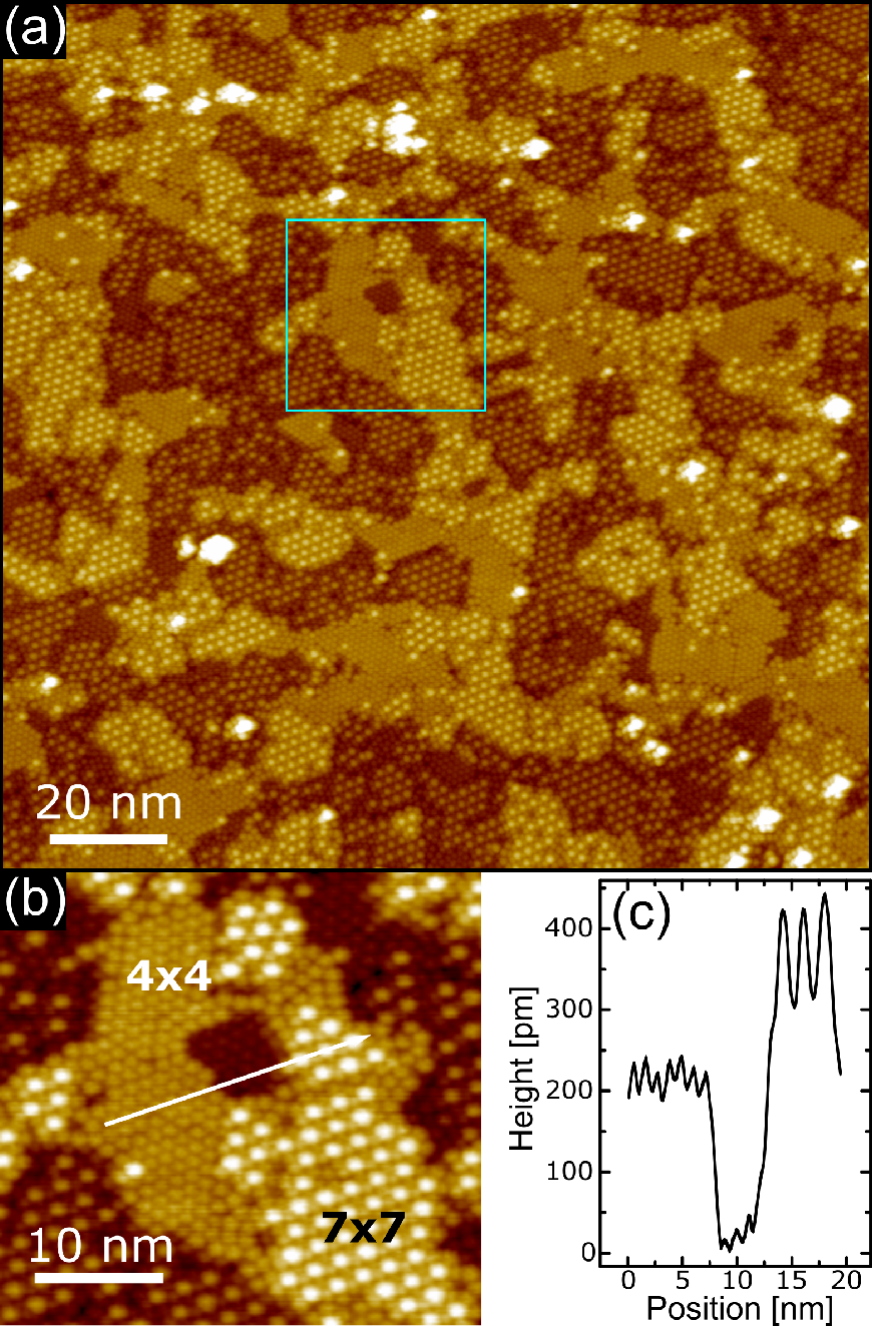
STM topography of a full C_60_ monolayer on Ni(111). (a) 1 ML C_60_ grown at RT and annealed at *T* = 400 °C for 5 min, with tunneling parameters Δ*V* = 1.5 V, *I* = 200 pA. The image size is 150 × 150 nm^2^. (b) Blow-up of panel a (region marked in light blue): image size 35 × 31 nm^2^; tunneling parameters Δ*V* = 1.5 V, *I* = 200 pA. (c) Line profile along the white arrow drawn in panel b.

Figure 3a displays a topographic image obtained after the deposition of about 1 ML of C_60_ at RT and subsequent annealing at *T* = 400 °C for 5 min. The surface is now characterized by many locally ordered small domains. While some of them show the already observed (4 × 4) phase, the surface is also characterized by the presence of regions in which fullerenes form a (7 × 7) overlayer. Panel b of Figure 3 shows a magnified image of a region from panel a in which it is possible to see that the (7 × 7) periodicity is due to molecules with an apparent height higher than that of the wetting layer. A line profile taken across two (4 × 4) domains and shown in Figure 3c reveals again a step height of about 200 pm, indicating that the different regions belong to distinct Ni(111) layers and thus confirming that the adsorption of C_60_ followed by annealing induces mass transport in the surface layer of the substrate and results in a roughening of the C_60_/Ni(111) interface.

**Figure 4:**
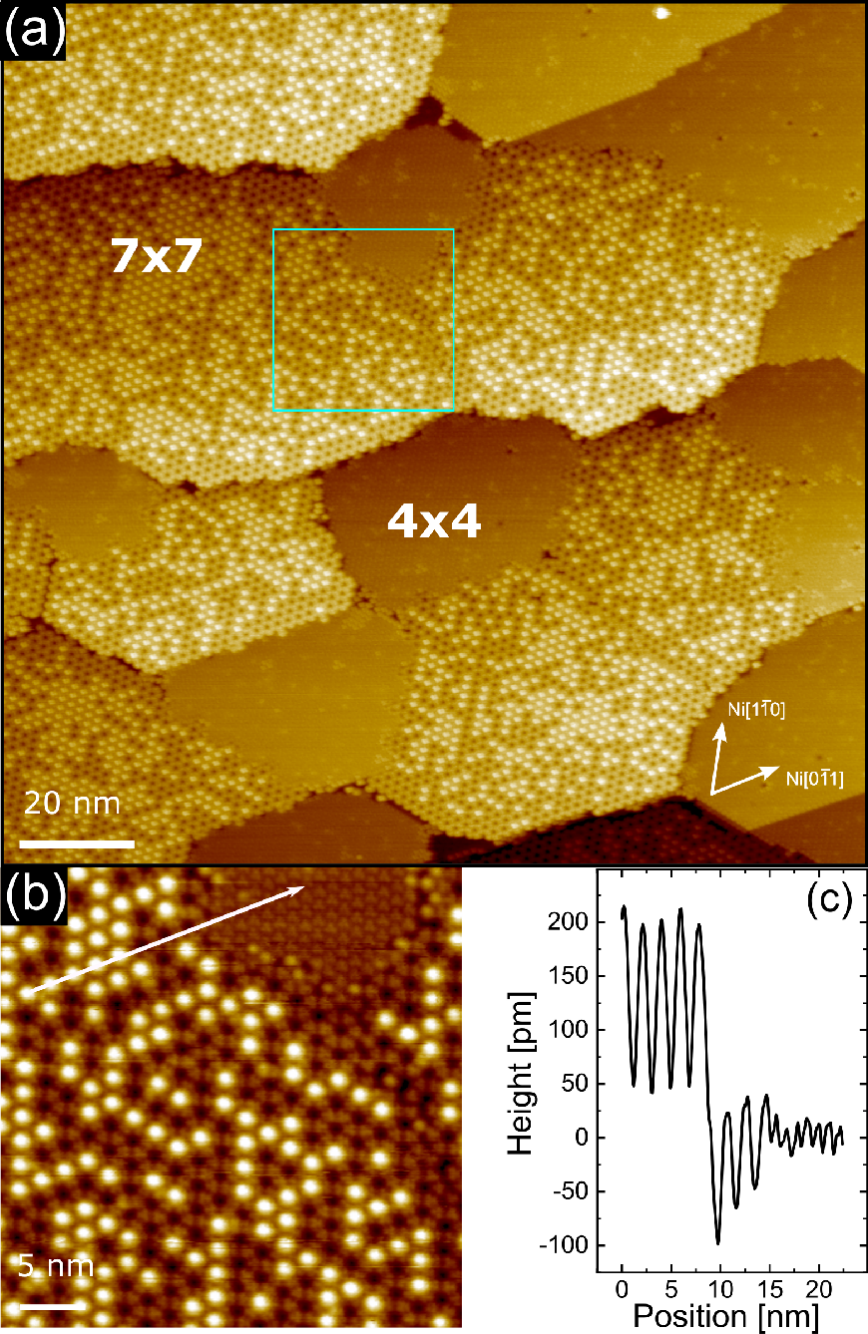
STM topography of a full C_60_ monolayer on Ni(111). (a) 1 ML C_60_ grown at RT and annealed at *T* = 400 °C for 10 min, with tunneling parameters Δ*V* = 1.5 V, *I* = 200 pA. The image size is 150 × 150 nm^2^. (b) Blow-up of panel a (region marked in light blue): image size 35 × 35 nm^2^; tunneling parameters Δ*V* = 1.5 V, *I* = 200 pA. (c) Line profile along the white arrow drawn in panel b.

A further annealing of the C_60_ monolayer at *T* = 400 °C for 5 min (total annealing time of 10 min) increases the order of the surface, which is now characterized by large terraces, as shown in Figure 4a. Here, the molecular layer is clearly seen to be formed by large (7 × 7) and (4 × 4) domains, with edges oriented along equivalent ⟨110⟩ directions of the Ni(111) substrate. A further effect induced by the prolonged annealing is the desorption of about one-half of the protruding C_60_ molecules, revealing that the (7 × 7) domains are formed by a honeycomb lattice of molecules. Figure 4b reports a magnified image where the (4 × 4) and (7 × 7) regions are both present. The topographic profile drawn along the white arrow, and reported in Figure 4c, helps in observing that the molecules forming the (4 × 4) domains and those belonging to the honeycomb network are accommodated on the same Ni(111) surface layer; therefore the prolonged annealing induces a smoothening of the C_60_/Ni(111) interface. On the other hand, the bright molecules of the (7 × 7) domain protrude by about 200 pm over the honeycomb lattice, suggesting that the uppermost molecules are partially embedded in the hollow sites.

**Figure 5:**
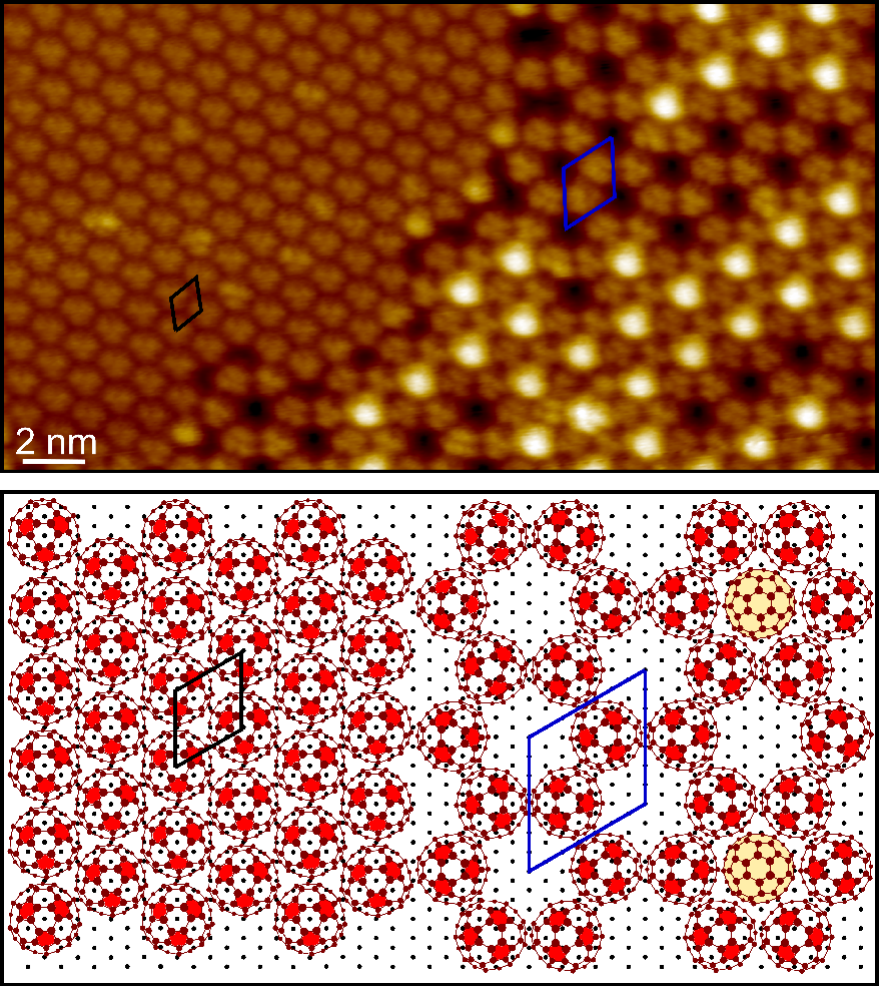
Highly resolved STM image acquired on a region in which the (4 × 4) and (7 × 7) phases coexist (tunneling parameters Δ*V* = 1.5 V, *I* = 500 pA). The black and blue parallelograms indicate the unit cell of the (4 × 4) and (7 × 7) phases, respectively. The image size is 28 × 15 nm^2^. The bottom panel reports a schematic model of the C_60_ adsorption geometry inferred from the STM measurements. The red colored pentagons represent the bright lobes experimentally observed inside the molecules. Two second-layer molecules are also added for exemplifying their in-plane positioning in the (7 × 7) phase. Note that they are uniformly colored in yellow, as the images do not allow resolving the internal electronic distribution.

Figure 5 displays a highly resolved STM image acquired at the boundary between a (7 × 7) and a (4 × 4) region. Remarkably, the molecular orbitals of each C_60_ molecule are clearly resolved, thus allowing one to establish the molecular orientation with respect to the substrate. Considering that in STM empty states images of C_60_, the pentagons of the cage structure appear as bright lobes,^37,38^ we conclude that in both domains the fullerene orientation is the one schematically reported in Figure 5b. It is significant that the molecular orientation of the (4 × 4) region, as inferred from our STM measurements, nicely corresponds to that obtained from the ab initio simulations discussed in ref (22). In particular, C_60_ adsorbs with a hexagonal face parallel to the Ni(111) surface and oriented like the hexagonal arrangement of atoms on the substrate surface.

As mentioned in the Introduction, the (4 × 4) phase was already observed for C_60_ overlayers on Ni(111), while the (7 × 7) phase was not previously reported. However, C_60_ deposited on Al(111) forms an overlayer with (6 × 6) periodicity very similar to the (7 × 7) phase observed on Ni(111); therefore it is useful to recall the main features of the C_60_/Al(111) interface.^39^ In particular, both systems are characterized by the formation of a honeycomb fullerene lattice, over which single C_60_ molecules are accommodated; the latter are imaged in STM measurements with an apparent height larger than that of the neighboring molecules. As highlighted in ref (19), the raised molecules are bound to Al ad-dimers present in the interstices of the C_60_ overlayer, which in turn are formed by the removal of single Al atoms from the Al(111) surface layer, induced by the adsorption of C_60_ forming the honeycomb lattice. In this frame, the topmost molecules are not just physisorbed onto the honeycomb lattice but form strong covalent bonds with the metal. In the (7 × 7) C_60_/Ni(111) a similar scenario might occur, even if further investigations are needed to confirm this hypothesis.

**Figure 6:**
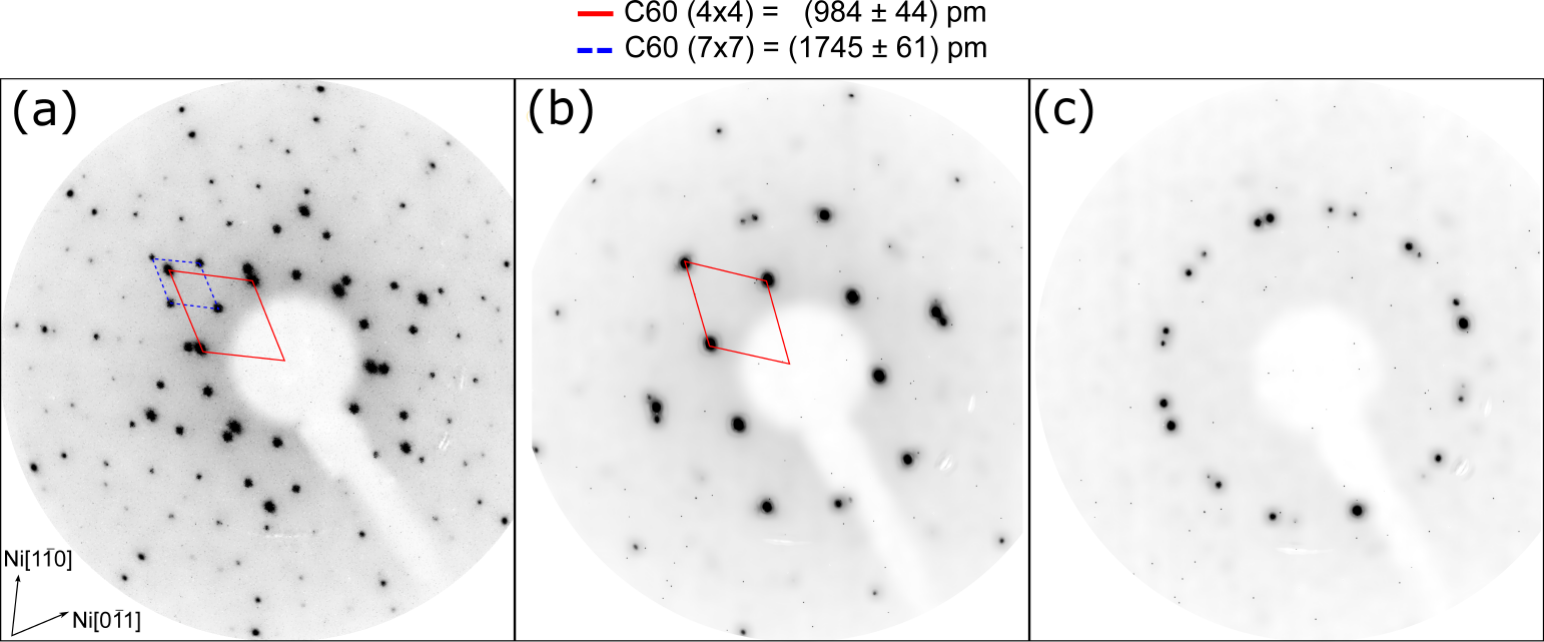
(a) LEED pattern of 1 ML C_60_/Ni(111) annealed at *T* = 400 °C for 10 min. (b) LEED pattern acquired after further 20 min of annealing at *T* = 400 °C. (c) LEED pattern acquired after molecular desorption, resulting in a cabide-covered Ni(111) surface. The unit cells for the (4 × 4) and (7 × 7) phases are indicated. The lattice constants calculated from LEED measurements are reported on top. The primary electron beam energy is 50 eV for all patterns.

The (7 × 7) phase eventually disappears after prolonged annealing of the sample, as shown in Figure 6. Panel a displays the LEED pattern acquired after deposition of 1 ML of C_60_ at RT and annealing at *T* = 400 °C for 10 min. Both phases coexist on the surface, in agreement with the STM measurements. The unit cells in the reciprocal space and their corresponding lattice units in the real space are also reported in Figure 6. The diffraction pattern shown in panel b was acquired on the same sample annealed at *T* = 400 °C for further 20 min. After this treatment, only the (4 × 4) periodicity is visible, while the (7 × 7) superstructure disappeared. Besides the spots due to the (4 × 4) C_60_ film, further faint diffraction features are visible. These extra spots are ascribed to the surface reconstruction induced by Ni carbides,^40^ indicating that the annealing induces the desorption of a fraction of the C_60_ film and the presence of uncovered substrate regions, as also confirmed by STM measurements (not shown). If the annealing time is further extended, all the molecules eventually desorb from the Ni(111) surface, leaving a carbide-covered Ni(111) surface. A LEED pattern of such reconstructed surface is reported in Figure 6c. The presence of the nickel carbides is mainly due to the migration of carbon impurities from the Ni bulk to the surface,^41^ as testified by the fact that even the annealing of the bare Ni(111) stabilizes the carbide reconstruction. However, we cannot exclude that a small fraction of C_60_ molecules dissociate during the annealing process, partially contributing to the Ni carbide formation.

In conclusion, the experimental observations suggest that, at low coverage, only the (4 × 4) is stable (see also Figure 2), while for a full monolayer both phases can self-assemble.

The formation mechanism of the (7 × 7) superstructure and its metastability deserve further discussion. A possible explanation of the fact that the (7 × 7) phase is observed only when the C_60_ molecules completely cover the Ni (111) surface, while for submonolayer coverages the (4 × 4) superstructure is solely present, might reside in the different kinetics of formation of the two phases. In both of them, the adsorption of C_60_ molecules proceeds via the removal of Ni atoms from the surface layer: in the (4 × 4) phase, the molecules simply adsorb inside the depressions created by the removed substrate atoms; in the (7 × 7), instead, one-third of the molecules (the protruding ones) must bind to Ni ad-dimers, similar to the C_60_/Al(111) case mentioned above. At high temperatures (in our case 400 °C), those ad-dimers are very mobile and unstable. Therefore, we expect that only a small fraction of them contribute to the formation and stabilization of the (7 × 7) superstructure. In this respect, only at high molecular coverages is the density of ad-dimers bound to molecules large enough to promote the formation of the (7 × 7) phase.

We also noticed that the (7 × 7) phase is metastable, in the sense that after a long enough annealing it disappears, leaving the surface covered by the (4 × 4) molecular superstructure, which remains stable up to higher temperatures. We argue that the lower stability of the (7 × 7) phase, compared to the (4 × 4) phase, can be justified by the fact that C_60_ molecules in the former are bound to only two atoms of Ni, while in the latter they are embedded between the first and the second layer of the substrate, thus having a larger coordination with the substrate atoms. In particular, we observe that a first, short heating typically induces the desorption of the molecules located in the cavities of the honeycomb lattice of the (7 × 7) phase. Apparently, this desorption destabilizes the (7 × 7) superstructure and favors its annihilation after additional annealing steps.

A different interpretation, based on energy considerations, can also be pondered. During the annealing process, the interaction between the C_60_ molecules and the sample surface likely leads to the displacement of a fraction of Ni atoms from the topmost layer, leaving a rough surface that exposes two layers and which should be characterized by several kinks. The (7 × 7) structure could be energetically favored because the highest molecules atop the cavities adsorb on bare regions of the Ni(111) surface (as also suggested by the fact that they protrude about 200 pm with respect to the surrounding molecules; see Figure 4), while the C_60_ molecules forming the honeycomb lattice decorate the kink sites. In this frame, a higher density of C_60_ present on the surface before the annealing process is expected to promote the creation of a high number of kinks and undercoordinated sites, favoring the stabilization of the (7 × 7) phase. Clearly, both thermodynamic and kinetic mechanisms could contribute to the formation of the new phase.

**Figure 7:**
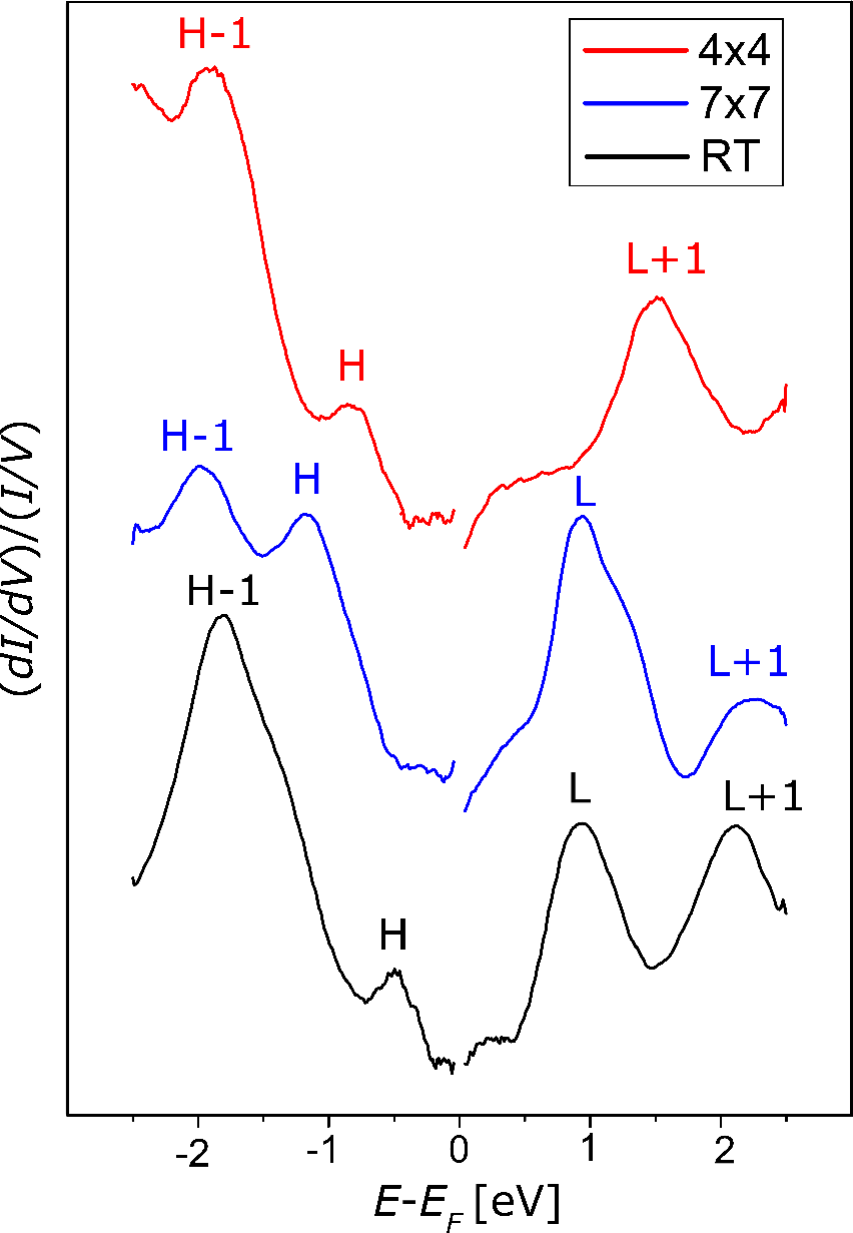
STS spectra acquired on C_60_/Ni(111) deposited at RT before annealing (bottom black spectrum), on (7 × 7) regions (middle blue spectrum), and on (4 × 4) regions (top red spectrum) obtained by annealing the as-grown sample at 400 °C for 10 min. Each curve is the average of several tens of spectra and is normalized to the respective total conductance *I*/*V*. The curves are displayed with a vertical offset for helping in visualizing the different features.

Figure 7 displays the STS spectra acquired on C_60_ deposited at RT (bottom spectrum) and on the annealed films, on either the (7 × 7) (middle spectrum) or the (4 × 4) (top spectrum) domains. In all cases, the curves are obtained by averaging several measurements on extended spatial regions, including at least a whole unit cell. In each spectrum, broad peaks associated with molecular orbitals are evident in both the empty (positive energy) and filled (negative energy) electronic states. The broadening of the molecular orbitals has been observed also for C_60_ adsorbed on other transition metal surfaces, like Fe and Co.^8,24^ This phenomenon is due to the hybridization of C_60_*p* orbitals and substrate *d* states. Here, we provide evidence that the structural details of the C_60_/Ni(111) interface strongly influence the electronic properties of the molecules.

The films deposited at RT exhibit two features at −0.5 eV and −1.8 eV, which can be associated with the HOMO (H) and HOMO–1 (H–1) molecular orbitals, respectively. On the empty states side of the spectrum, the LUMO (L) and LUMO+1 (L+1) orbitals are located at 0.9 and 2.1 eV. It is interesting to notice that the H–L gap, which corresponds to the difference between the ionization potential and the electron affinity, is about 1.4 eV, quite smaller than the H–L gap of 4.95 eV characteristic of the isolated C_60_ molecule.^42,43^ This behavior is ascribed to the presence of a metal substrate, which provides screening of the electron–electron interactions within each C_60_ molecule and leads to the reduction of the energy separation between the HOMO and LUMO peaks observed in STS.^44^

On the (7 × 7) sample, the molecular resonances are characterized by a width comparable with that of the RT sample. The larger H–L gap (about 2 eV) suggests a less efficient screening provided by the substrate with respect to the RT case. The interpretation of the spectrum is complicated by the fact that the (7 × 7) unit cell hosts two nonequivalent molecules, i.e., those in the honeycomb lattice and those in the cavities of the latter. For instance, the L peak shows an asymmetric shape suggesting the superposition of two different features, which we believe can be ascribed to different molecules rather than to different states.

The electronic structure of the (4 × 4) phase is remarkably different from that measured on (7 × 7) and RT samples. In particular, the shape of the L peak is not well-defined, suggesting a very strong hybridization with the electronic states of the substrate, likely promoted by the fact that the molecules are partially embedded on the topmost Ni(111) layer. This is in good accordance with the discussion about the metastability of the (7 × 7) phase reported above. Interestingly, the observation of a remarkable hybridization of L with the states of the substrates is in excellent agreement with the calculations performed in ref (22) about the electronic structure of the (4 × 4) phase.

## 4 Conclusions

We have reported a structural and electronic characterization of the C_60_/Ni(111) interface. Two ordered phases, forming either a (4 × 4) or a (7 × 7) superstructure, are found to coexist for a coverage of about 1 ML, while in the sub-ML regime only the (4 × 4) phase is stable. The STS spectra reveal a significant hybridization between the electronic states of the Ni(111) substrate and the molecular orbitals, whose strength depends on the stabilized phase.

Even if the present work does not present magnetic characterizations, we have underlined that interfaces between molecular layers and magnetic interfaces are the building blocks of organic spintronics. In this rapidly developing research field, one of the main challenges is that of enhancing the interface coupling that, by forming spin-polarized hybrid interface states,^14^ can turn on specific mechanisms, such as spin filtering effects, suitable for the development of innovative devices. In this respect, we remark that recent calculations have definitely highlighted the fact that the structural and electronic details of the molecular adsorption onto a magnetic substrate have a central role in defining the spin polarization of the interface states and thus, in perspective, a potential device performance.^45,46^

Several future developments and perspectives can be identified for the investigation of the C_60_/Ni interface. First of all, while phase (4 × 4) has been investigated by ab initio simulations, the (7 × 7) overlayer deserves further theoretical analysis. In the second place, the predicted correlation between the interface structure and the magnetic coupling must be experimentally analyzed with the help of spin-resolved techniques.
